# Nutrient deprivation induces α-synuclein aggregation through endoplasmic reticulum stress response and SREBP2 pathway

**DOI:** 10.3389/fnagi.2014.00268

**Published:** 2014-10-08

**Authors:** Peizhou Jiang, Ming Gan, Wen-Lang Lin, Shu-Hui C. Yen

**Affiliations:** Department of Neuroscience, Mayo Clinic College of MedicineJacksonville, FL, USA

**Keywords:** Parkinson’s disease, α-synuclein, ER stress, autophagy, aggregation, SREBP2, nutrient deprivation

## Abstract

Abnormal accumulation of filamentous α-synuclein (α-syn) in neurons, regarded as Lewy bodies (LBs), are a hallmark of Parkinson disease (PD). Although the exact mechanism(s) underlying LBs formation remains unknown, autophagy and ER stress response have emerged as two important pathways affecting α-syn aggregation. In present study we tested whether cells with the tetracycline-off inducible overexpression of α-syn and accumulating α-syn aggregates can benefit from autophagy activation elicited by nutrient deprivation (ND), since this approach was reported to effectively clear cellular polyglutamine aggregates. We found that nutrient deprivation of non-induced cells did not affect cell viability, but significantly activated autophagy reflected by increasing the level of autophagy marker LC3-II and autophagic flux and decrease of endogenous α-syn. Cells with induced α-syn expression alone displayed autophagy activation in an α-syn dose-dependent manner to reach a level comparable to that found in non-induced, nutrient deprived counterparts. Nutrient deprivation also activated autophagy further in α-syn induced cells, but the extent was decreased with increase of α-syn dose, indicating α-syn overexpression reduces the responsiveness of cells to nutrient deprivation. Moreover, the nutrient deprivation enhanced α-syn aggregations concomitant with significant increase of apoptosis as well as ER stress response, SREBP2 activation and cholesterolgenesis. Importantly, α-syn aggregate accumulation and other effects caused by nutrient deprivation were counteracted by knockdown of SREBP2, treatment with cholesterol lowering agent—lovastatin, or by GRP78 overexpression, which also caused decrease of SREBP2 activity. Similar results were obtained from studies of primary neurons with α-syn overexpression under nutrient deprivation. Together our findings suggested that down-regulation of SREBP2 activity might be a means to prevent α-syn aggregation in PD via reducing cholesterol levels.

## Introduction

Parkinson disease (PD) is a neurodegenerative disorder characterized pathologically by loss of dopaminergic neurons in the substantia nigra and abnormal accumulation of α-synuclein (α-syn) as filamentous aggregates in neuronal perikarya and processes referred to as Lewy bodies (LBs) and Lewy neurites (LNs), respectively (Cookson, 2005; Lippa et al., 2007). Genetic studies have shown a link between PD and multiplication of *SCNA* gene encoding α-syn (Singleton et al., 2003; Chartier-Harlin et al., 2004; Ibáñez et al., 2004). Although the exact mechanisms underlying the formation of LBs and LNs remain unclear, a lot of attention has been drawn to identify etiological factors/pathways affecting α-syn aggregation either directly or indirectly. In this regard autophagy and unfolded protein response (UPR) have emerged as two pathways of importance (Hoozemans et al., 2007; Pan et al., 2008; Lynch-Day et al., 2012; Mercado et al., 2013). UPR is triggered by a variety of conditions that disturb protein folding in endoplasmic reticulum (ER; Zhang and Kaufman, 2006). Studies of human brain specimens from PD affected cases have shown the occurrence of UPR at higher magnitudes than those in counterparts from age-matched normal subjects (Hoozemans et al., 2007). It has been demonstrated before and verified in present study that ER stress can be elicited by overexpression of α-syn (Cooper et al., 2006; Jiang et al., 2010; Bellucci et al., 2011), and cultured cells with induced α-syn expression accumulate α-syn oligomers (Ko et al., 2008; Jiang et al., 2010, 2013b). Moreover, exposure to ER stress inducers increased further the levels of α-syn assemblies (Jiang et al., 2010).

Autophagy is a physiological process by which cells remove useless organelles and degrade proteins through lysosomes (Reggiori and Klionsky, 2002). Dysfunction of autophagy impedes the degradation of many proteins including α-syn, and can result in accumulation of different products of α-syn (Pan et al., 2008; Klucken et al., 2012). Therefore, activation of protein degradation pathways such as autophagy has been considered as a strategy to reduce the accumulation of intracellular α-syn oligomers, some of which may be cytotoxic (Winner et al., 2011). Cell-based studies have shown that autophagy can be activated by nutrient deprivation (ND)—elimination of different nutrient factors such as B27 supplement (Young et al., 2009), amino acid (Munafó and Colombo, 2001; Ghislat et al., 2012), glucose deprivation (Marambio et al., 2010) or treatment with various agents including rapamycin (Chong et al., 2012; Graziotto et al., 2012). It has been reported that culturing neuronal cells in medium without B27 supplementation is a robust method for autophagy activation without causing rapid cell death (Young et al., 2009). This approach was shown in previous studies to protect neuronal cells from toxicity caused by expressing polyglutamine-expanded proteins (Young et al., 2009). Since α-syn is significantly increased in aging brain (Jellinger, 2004; Li et al., 2004; Chu and Kordower, 2007) and malnutrition is promoted by aging process (Hickson, 2006), we wonder whether or not B27 deprivation (hereafter called nutrient deprivation) have any effect on α-syn level. To address this issue, we used a neuronal cell model regarded as 3D5 (Takahashi et al., 2007). Cells of this model are inducible to express human wild-type α-syn via the tetracycline-off (Tetoff) mechanism and can persistently accumulate α-syn upon retinoic acid (RA)-elicited differentiation (Ko et al., 2008; Jiang et al., 2010).

We found that in 3D5 cells without α-syn induction (i.e., expressing only endogenous), nutrient deprivation significantly activated autophagy reflected by increasing the level of autophagy marker LC3-II and autophagic flux, leading to decrease of endogenous α-syn. Interestingly, in sibling cultures with α-syn induction, overexpressed α-syn activated autophagy in a dose dependent manner. Nutrient deprivation also activated autophagy further in α-syn induced cells, but the extent was decreased with increase of α-syn induction duration, indicating α-syn overexpression reduces the responsiveness of cells to nutrient deprivation. Moreover, tremendous α-syn aggregation concomitant with significant cell apoptosis were induced. These findings prompted us to identify the key gene(s) and pathway(s) related to the above changes.

## Materials and methods

### Cell models

Human wild-type α-syn transfectant 3D5 was derived from human neuroblastoma BE2-M17D cell line (Takahashi et al., 2007). It expresses α-syn and displays neuronal phenotypes upon TetOff induction and incubation with RA, respectively (Ko et al., 2008). For maintenance, cells were cultured in DMEM/10% fetal bovine serum with 2 µg/mL Tet at 37°C and 5% CO_2_. Cells were seeded at the density of 1.0 × 10^5^ cells/well in 6-well plates for biochemical analysis, 2 × 10^4^ cells/well on coverslips in 24-well plates for neutral lipid or cholesterol staining, and 1 × 10^4^ in 48-well plates (Bellco Glass Inc., Vineland, NJ) for spectrophotometric assay, respectively. To induce and maintain cells under differentiated condition, neurobasal medium (Invitrogen, Carlsbad, CA) supplemented with 2% B-27 (Invitrogen), 2 mM L-glutamine (Sigma Aldrich) and 10 µM RA (Sigma-Aldrich, St Louis, MO) were used. To initiate nutrient deprivation we excluded B27 from the Neurobasal medium.

For primary cultures, cortical/hippocampal neurons from embryonic FVB mice were seeded on poly-D- lysine (Sigma) coated 6- and 48-well plate at about 0.8 × 10^6^ and 1 × 10^5^ cells per well, respectively. Experimental protocols for primary cultures were the same as those reported previously (Kivell et al., 2001). After 3 days of culturing, neurons were infected with lentivirus carrying genes or shRNAs of interests. 7 days post viral infection, neurons were subjected to nutrient deprivation for 24 h.

### Animals

All animal procedures were approved by the Mayo Clinic Institutional Animal Care and Use Committee (IACUC) and were in accordance with the National Institute of Health Guide for the Care and Use of Laboratory Animals (NIH Publications No. 80–23) revised 1996.

### Lentiviral plasmids and virus preparation

Lentiviral plasmid carrying α-Syn [EX-G0543-Lv105], GRP78 [EX-T3592-Lv105], LAMP1-eCFP [Ex-T8016-Lv154] and LC3-mCherry [EX-Z5296-Lv111] were purchased from Genecopoeia, and shRNAs of human and mouse SREBP2 from Sigma Aldrich, respectively. The preparation of Lentivirus carrying genes of interest or shRNA were the same as described previously (Jiang et al., 2013a).

### Antibodies

Antibodies were mouse monoclonal antibodies to α-Syn (BD Biosciences), α-synuclein (LB 509, Santa Cruz Biotechnology), GRP78 (BD Biosciences), Lamp2(Santa Cruz Biotechnology), MAP2 and β-actin (Sigma Aldrich), total EIF2α (T-EIF2α, Cell Signaling) as well as rabbit polyclonal antibodies to total mammalian target of rapamycin (T-mTOR, Cell Signaling), Ser2448 phosphorylated mTOR (p-mTOR, Cell Signaling), p62 (Cell Signaling), Beclin-1 (Cell Signaling), Caspase 12 (Cell Signaling), SREBP2 (Cayman Chemical), SREBP1 (Pierce), cleaved Caspase-3 (Cell Signaling), phosphorylated EIF2α (pEIF2α, Invitrogen), GADD153/CHOP10 (Sigma Aldrich) and LC3 (Novus Biologicals).

### Western blot analysis

Cell cultures were harvested and centrifuged at 200 × g for 15 min. Cell pellets were lysed in RIPA buffer (Cell signaling) for analysis of SREBP2, SREBP1 and MES buffer (Jiang et al., 2013b) for analysis of other proteins. All lysis buffers were supplemented with phosphatase inhibitors. Upon lysis cell lystates were mixed with Tricine-SDS sample buffer (Invitrogen) and 2% β-mercaptoethanol, boiled for 5 min and resolved by SDS-PAGE using 10–20% Tris/Tricine gel (Bio-rad). Precision plus protein standards (Bio-Rad) were include in SDS-PAGE as reference markers. After gel elctrophoresis, proteins were transferred onto nitrocellulose for further probing with antibodies against proteins of interest. Western Lightning Plus ECL (PerkinElmer) or ECL™ Prime Western Blotting Detection Reagent (Fisher Scientific) were used for visualization of protein immunoreactivities. Immunoreactivity of β-actin was used as loading controls.

### Quantitative real-time PCR

Four sets of 3D5 cells with RA-elicited neuronal differentiation were used. They included cells with and without α-syn induction for 10 days and corresponding sibling cultures under nutrient deprivation. Total RNA from each sample was isolated using RNeasy Mini Kit (Qiagen), and 3 µg were used to synthesize cDNA using SuperScript^®^ III First-Strand Synthesis System (Invitrogen). The reaction mixtures were subjected to quantitative real-time PCR to determine the transcript level of α-syn and LC3, respectively. Primers for α-syn, LC3 and β-actin were sc-29619-PR, sc-43390-PR and sc-108069-PR from Santa Cruz Biotechnology, respectively. Triplicate reactions per sample were prepared using a 25-µl mixture containing Platinum SYBR Green qPCR Super Mix UDG (Invitrogen). The same analysis was performed with four samples from three set of experiments. All data were normalized to the endogenous β-actin transcripts. The PCR was run on the ABI 7900 and data analyzed using Software RQ Manager 1.2 (Applied Biosystems).

### Electron microscopy

To evaluate ultrastructural changes of 3D5 cells under nutrient deprivation, we used cells grown on Thermanox plastic coverslips (diameter, 13 mm; Nunc, Naperville, IL) in 24-well plates and process them for electron microscopy as previously reported (Jiang et al., 2010).

### Immunocytochemistry

Cells grown on cover slips were rinsed with phosphate buffered saline, fixed in 4% paraformaldehyde, and permeabilized with 0.1M Tris-buffered saline (TBS; pH 7.6) containing 0.5% Triton X-100 for 5 min. They were subsequently blocked with 3% goat serum in TBS, incubated with antibody SREBP2 and LB509 or MAP2 in TBS containing 1% goat serum overnight at 4°C then incubated for 1 h with goat anti-rabbit antibody conjugated with Alexa594 and goat anti-mouse conjugated with Alexa488. Immunolabeled cells were stained with nuclei stain DAPI (Invitrogen) for 10 min and observed by confocal fluorescence microscopy (Zeiss LSM 510, Carl Zeiss MicroImaging). Zen 2009 software was used for imaging and subsequent quantitation of nuclear SREBP2 fluorescence intensity.

### Confocal microscopy of live cells

Differentiated 3D5 cells without α-syn induction but co-infected with lentivirus carrying LAMP1-eCFP and LC3-mCherry were cultured in Lab-Tek™ Chambered Cover Glass System (4 well, Lab-Tek II). They were induced to express α-syn for 0, 3, 6, 9 and 12 days to obtain samples containing different levels of α-syn. Duplicate cultures were subjected to nutrient deprivation for 24 h. These live cells were examined by confocal microscopy (Zeiss LSM 510, Carl Zeiss MicroImaging) for evaluation of the expression and distribution of LAMP1-eCFP and LC3-mCherry.

### Quantification of LAMP1-eCFP and LC3-mCherry co-localization

Images of multiple fields from different experimental paradigms and at least three repeated experiments were captured. Each field has about five cells. Same setting of exposure time was used throughout the course of image acquisition. We used Zen 2009 software to evaluate the distribution of LAMP1-eCFP and LC3-mCherry. Pearson’s correlation coefficient and Mander’s overlap coefficient were used for assessing co-localization and compared for determination of the difference of autophagy flux between different groups.

### Neutral lipids and cholesterol staining *in situ* and fluorescence microscopy

Cells grown on coverslips were fixed in 4% paraformaldehyde for 30 min at room temperature (RT). They were then incubated for 30 min in LipidTox™ Green (HCS LipidTox™ Steatosis detection kits, Invitrogen), washed with PBS twice and stained with PBS containing Hoechst 33342 (0.5 µg/ml; Sigma Aldrich) for 10 min to detect neutral lipid and nuclei, respectively. For detection of cholesterol, cells were incubated in freshly prepared 50 µg/ml Filipin in PBS for 1 h at RT, followed by washing with PBS twice. Stained cells were mounted on glass slides with Prolong Antifade solution (Invitrogen) and imaged at RT using an Olympus DP70 camera attached to Olympus BX50 fluorescent microscope (Olympus) with a Lumen 200 Fluorescence Illumination System (Prior Scientific). The LipidTOX™ Green signals were visualized with 470/40-nm band-pass excitation filter and a 525/50-nm band-pass emission filter. The Filipin signal was visualized with 360/40-nm band-pass excitation filter and a 470/50-nm band-pass emission filter. Images were processed using DPController and DPManager software (Olympus). To quantify intracellular neutral lipids and cholesterol, outlines of the whole cell and nucleus were manually drawn and fluorescence signals emitted by LipidTOX™ Green or Filipin were measured using ImageJ (National Institutes of Health). Intracellular neutral lipids and cholesterol contents were presented by subtracting fluorescence signals emitted by the nucleus from that of whole cell. Relative levels of intracellular neutral lipids and cholesterol were obtained by normalizing the readouts derived from nutrient-deprived cells against those of control cells, which were arbitrarily defined as 1. Five fields per cover glass/sample and more than 100 cells were randomly imaged and analyzed. Data derived from three samples per experimental condition were used for statistical analysis.

### Intracellular cholesterol content measurement

Cellular levels of free cholesterol were measured using an Amplex Red cholesterol assay kit according to manufacturer’s instructions (Invitrogen). In brief, cells were lysed and incubated with cholesterol oxidase, horseradish peroxidase, and Amplex red in the absence or presence of cholesterol esterase. The amount of cholesterol was determined indirectly by measuring resorufin absorbance at 560 nm. The values were normalized to the total cellular protein levels, which were determined with a Bicinchoninic Acid (BCA) protein assay kit (Thermo Fisher Scientific).

### Cell viability

We used Calcein AM assay to assess cell viability. Cultures of 3D5 cells or cortical/hippocampal neurons were incubated with 2 µM calcein AM (Invitrogen) in balanced salt solution for 30 min at RT in the dark. Fluorescence signals emitted from live cells by esterase-metabolized calcein were measured at 495 nm (excitation)/530 nm (emission) using a Spectra Max M4 and Soft Max Protein 4.6 software (Molecular Devices, Sunnyvale, CA). All measurements were performed in triplicate from three experiments.

### Cathepsin D activity assay

The activity of Cathepsin D was analyzed using Cathepsin D Activity Fluorometric Assay Kit (BioVision). Cells (about 1 × 10^6^) were lysed in 200 µl of chilled CD Cell Lysis Buffer followed by 5 min of centrifugation at top speed. The supernatant was collected and BCA Assay was performed for measurement of protein concentration. The equal amount of protein from each group was loaded into a 96-well plate and incubated with the master assay mix (50 µl/well) at 37°C for 1 h. Samples were read in a fluorometer equipped with a 328-nm excitation filter and 460-nm emission filter.

### Statistical analysis

Data from at least three sets of independent experiments were analyzed by one-way Anova with Dunnett’s *post hoc* test or Student’s *t*-test for comparison of groups >3 and = 2, respectively, to determine statistical significance.

## Results

### Macroautophagy is predominantly responsible for α-syn degradation in 3D5 cells upon nutrient deprivation

**Figure 1:**
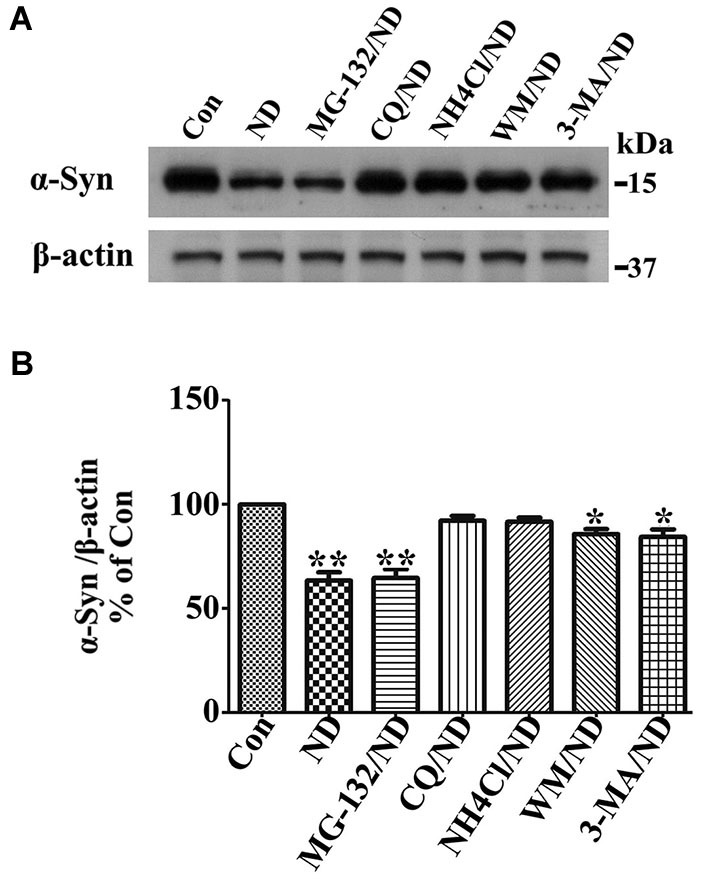
**Macroautophagy is predominantly responsible for α-syn degradation in 3D5 cells upon nutrient deprivation (ND)**. RA-elicited differentiated 3D5 cells bearing only endogenous α-syn, were respectively subjected to B27 deprived media with or without addition of different inhibitors MG-132, CQ, NH4Cl, 3-MA and WM for 24 h. The α-syn level in cells without nutrient deprivation (Con) was set as 100%, which was the initial level of α-syn for degradation in the following 24 h under ND. **(A)** The degradation of α-syn was evaluated and compared by western blotting of cell lysates. **(B)** Bar graph summarized the data from three independent experiments. Error bars represent standard error of the mean (* *p* < 0.05, ** *p* < 0.01, comparing to Con).

Previous studies have shown that wild type α-syn can be degraded by different pathways including proteasome, chaperone-mediated autophagy (CMA) and macroautophagy in neuronal cells (Webb et al., 2003; Vogiatzi et al., 2008). We have previously identified that macroautophagy is the predominant pathway for α-syn degradation in 3D5 cells in complete medium (data no shown). Here, we wondered if this is still the case when cells are maintained under nutrient deprivation condition which might activate both CMA and macroautophagy (Cuervo et al., 2004). For this purpose, we followed previously established method (Zhang et al., 2010; Jiang et al., 2013b) for this particular Tetoff cell system in which the concentration of different inhibitors for blocking certain degradation pathways have already been optimized. It is worth noting that the cells we used here are only with endogenous α-syn because those with overexpressed α-syn are vulnerable to inhibitors under nutrient deprivation. Cells without α-syn induction were differentiated for 10 days and then maintained in B27 deprived media with or without addition of different inhibitors to respectively block the function of proteasome (MG-132), lysosome (chloroquine (CQ) and NH4Cl) and the formation of autophagosome (3-MA and wortmannin (WM)) for 24 h, the degradation of α-syn was evaluated and compared by western blotting of cell lysates (Figure 1). The α-syn level in cells without nutrient deprivation (Con) was set as 100%, which was the initial level of α-syn for degradation in the following 24 h under nutrient deprivation. Without treatment of any inhibitor, α-syn level left in cells after 24 h of degradation under nutrient deprivation was about 63%. In contrast, (1) in cell treated with MG-132, α-syn level was reduced to about 63%, indicating that inhibition of proteasome pathway has no effect on α-syn degradation; (2) in cells treated with CQ and NH4Cl, α-syn level was reduced to about 99% and 98%, which did not show statistically significant difference comparing to initial level, indicating an almost complete block of α-syn degradation; and (3) in cells treated with 3-MA and WM, α-syn level was reduced to about 97% and 96%, which showed statistically significant difference comparing to initial level, indicating a predominant but not complete block of α-syn degradation. Therefore, we conclude that under nutrient deprivation, (1) α-syn in 3D5 cells is preferentially degraded through lysosome not proteasome; (2) macroautophagy is still the dominant pathway responsible for α-syn degradation through lysosomes; and (3) chaperon mediated autophagy or other pathway, if there is any, should be responsible for less than 1% of total α-syn. Accordingly, we will only focus on macroautophagy (hereafter called autophagy) to study degradation- related changes of α-syn level under nutrient deprivation in present study.

### Differential effects of nutrient deprivation on autophagy activation in cells with vs. without a 10 day induced α-syn overexpression

**Figure 2:**
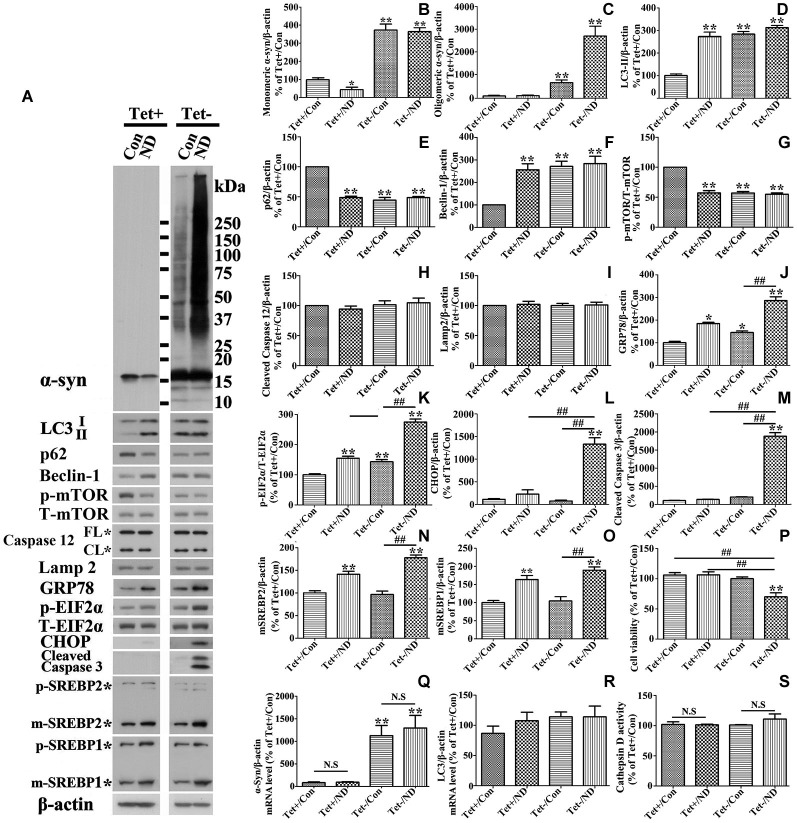
**Cells with 10 days of α-syn induction differ from those without induction in response to nutrient deprivation**. RA-elicited differentiated 3D5 cells with or without TetOff induction for 10 days, regarded as Tet (−) and Tet (+), were respectively subjected to nutrient deprivation for 24 h. Sibling cells without nutrient deprivation were used as Controls (Con). **(A)** Cell lysates were probed with antibodies to α-syn, LC3, p62, Beclin-1, p-mTOR, T-mTOR, Caspase 12, Lamp2, GRP78, p-EIF2α, T-EIF2α, CHOP, cleaved Caspase 3, SREBP1, SREBP2 and β-actin by western blotting. Molecular weight standards were included as references. Duplicate cultures were included for cell viability assessment. **(B–O)** Bar graphs summarized quantitative analysis of the immunoreactivities of interesting proteins from three independent experiments and normalized with β-actin or T-EIF2α immunoreactivities. The average values of Tet+/Con group were set as 100%. **(P)** Calcein assay showed that nutrient deprivation caused a significant lose of viability in cells overexpressing α-syn, but not in those expressing endogenous α-syn. **(Q,R)** Quantitative real-time polymerase chain reaction showed that nutrient deprivation did not change the transcripts level of α-syn and LC3 in 3D5 cells with and without α-syn overexpression (*p* > 0.05; *n* = 3). **(S)** The assay showed that nutrient deprivation did not change the activity of Cathepsin D in 3D5 cells with and without α-syn overexpression (*p* > 0.05; *n* = 3). Bar graph summarized the data from three independent experiments. Error bars represent standard error of the mean (* *p* < 0.05, ** *p* < 0.01, comparing to Tet+/Con; ^#^ *p* < 0.05, ^##^ *p* < 0.01, comparing subsets linked by line; N.S., no significant difference comparing subsets linked by line).

RA treated 3D5 cells with or without the TetOff induction for 10 days were maintained in nutrient deprived media for 24 h, and sibling cultures were kept in complete media (i.e., no deprivation) as controls. Lysates from cells were probed with antibodies to autophagy related markers LC3-II, p62, beclin-1, p-mTOR and T-mTOR by Western blotting. In the absence of nutrient deprivation, cultures with induced α-syn overexpression displayed significantly higher levels of LC3-II and beclin-1, and lower levels of p62 and p-mTOR, comparing to non-induced counterparts (Figures 2A,D,E,F,G), indicating the activation of autophagy through m-TOR pathway. Nutrient deprivation caused a significantly increased levels of LC3-II and beclin-1, and decreased level of p62 and p-mTOR in cells without α-syn induction, but did not affect the levels of the above markers in those overexpressing α-syn (Figures 2A,D,E,F,G). Therefore, cells with α-syn overexpression were not as responsive to nutrient deprivation in autophagy activation as those without. We assumed that such irresponsiveness might be due to the overexpressed α-syn which already activated autophagy to its maximum extent so that nutrient deprivation could not further enhance it.

### Effects of α-syn expression levels on LC3-II and autophagy flux in 3D5 cells with and without nutrient deprivation

To further confirm the aforementioned assumption, we first need to know whether different levels of α-syn expression affect autophagy differently in cultures under B27-deprived or non-deprived condition. To achieve different levels of α-syn expression, we used cultures with various durations (i.e., 0, 3, 6, 9 and 12 days) of Tetoff induction, since longer induction leads to more α-syn production (Takahashi et al., 2007; Ko et al., 2008; Jiang et al., 2013b).

Autophagy activation was monitored by measuring the level of LC3-II in cell lysates plus monitoring autophagic flux by image analysis of cultures expressing mCherry-tagged LC3 and eCFP-tagged LAMP1. This is in view of previous findings that increase of LC3-II level can be the result of either increase of autophagy flux or inhibition of fusion between autophagosome and lysosome, and that autophagic flux in different experimental paradigms can be compared by determining the extent of co-localization between LC3 and LAMP1 (Kimura et al., 2007; Korolchuk et al., 2011).

**Figure 3:**
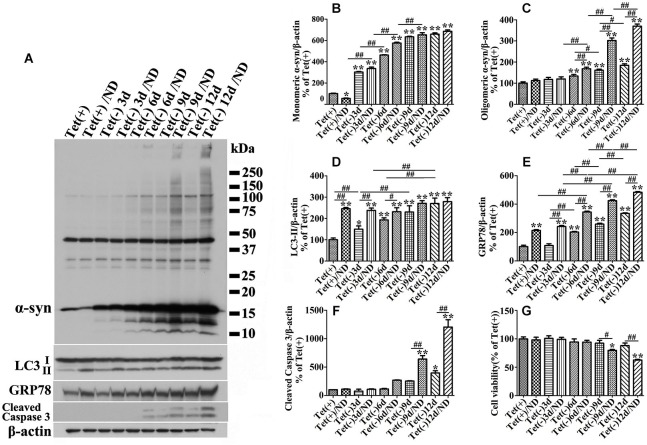
**Cultures expressing different levels of α-syn respond to nutrient deprivation differently in the expression of markers of ER stress response, autophagy and apoptosis**. 3D5 cells differentiated with RA were induced for 0, 3, 6, 9, 12 days to express different levels of α-syn before exposed to media with or without nutrient deprivation for 24 h. **(A)** Cell lysates were probed with antibodies to α-syn, LC3, GRP78, caspase 3 and β-actin for evaluation of α-syn aggregation, autophagy activation and ER stress response. Molecular weight standards were included as references. Duplicated cultures were included for cell viability assessment. **(B–F)** Bar graphs summarized the results of quantitative analyses of immunoreactivities of various proteins in cell lysates from three independent experiments with normalization against β-actin immunoreactivities. The average values of Tet (+) group were set as 100%. **(G)** Calcein assay showing nutrient deprivation caused a significant decrease of cell viability in cells with 9–12 days, but not shorter periods of α-syn induction. Error bars represent standard error of the mean (* *p* < 0.05, ** *p* < 0.01, comparing to Tet (+); ^#^ *p* < 0.05, ^##^ *p* < 0.01, comparing subsets linked by line, *n* = 3).

**Figure 4:**
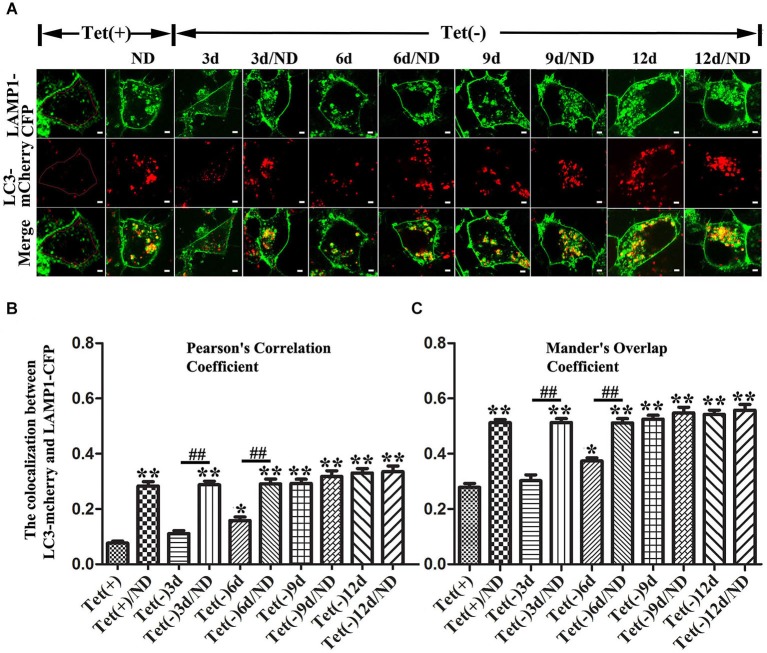
**Effects of different levels of α-syn expression on autophagic flux in 3D5 cells with and without nutrient deprivation**. 3D5 cells expressing LAMP1-eCFP and LC3-mCherry were cultured in Lab-Tek™ chambered cover glass system, induced to express α-syn for 0, 3, 6, 9 and 12 days, and maintained in media with or without nutrient deprivation for 24 h. **(A)** Confocal fluorescence microscopy of live cells showing the expression and distribution of LAMP1-eCFP and LC3-mCherry. An intact cell can be recognized by signals from LAMP1-eCFP expressed on cell membrane. Scale bars: 2 µm. Evaluation of autophagic flux based on analyses of **(B)** Pearson’s correlation coefficient and **(C)** Mander’s overlap coefficient. To avoid interference caused by LAMP1-eCFP signals from cell membrane, only cytoplasmic area was used for image analyses as shown in cell image of Tet (+) (area circled by red line) in **(A)** (*n* = 20; ** *P* < 0.01, * *P* < 0.05, comparing to Tet (+); ## *p* < 0.01, comparing subsets linked by line).

Our quantitative analyses of western blots of cultures lysates from cultures with induced α-syn expression and without nutrient deprivation revealed the increase of LC3II levels in an induction time (or α-syn dose) -dependent manner (Figures 3A,D). It is worth noting that 3 days of α-syn induction in 3D5 cells, which produced almost three fold of endogenous α-syn, already triggered significant increase of LC3II level (Figures 3A,B,D). In comparison to cells maintained in non-deprived media for up to 6 days, corresponding cultures with nutrient deprivation expressed significantly more LC3II. Small, but statistically insignificant differences in LC3II level were detected between nutrient-deprived and non-deprived cells with longer than 6 days of α-syn induction. Consistent with these findings were the results from confocal microscopy, demonstrating more intense fluorescence signals of LC3 and LAMP1 per cell in cultures with longer durations of induced α-syn expression (Figure 4A). Moreover, our analyses of Pearson’s correlation coefficient and Mander’s overlap coefficient of images derived from confocal microscopy revealed that most signals of LC3 co-localize with those of LAMP1 (Figures 4B,C), and that the value of these coefficients was significantly higher for cultures with up to 6 days of α-syn induction plus nutrient deprivation than non-deprived counterparts, indicating nutrient deprivation causes increase of autophagic flux in cells. However, such coefficients did not differ significantly between nutrient-deprived or non-deprived cells with 9 days or longer durations of induced α-syn expression, indicating a plateau is reached. Therefore, our results showed that cells with induced α-syn expression alone displayed autophagy activation in an α-syn dose-dependent manner; Nutrient deprivation also enhanced autophagy further in α-syn induced cells, but the extent was decreased with the increase of α-syn dose; Moreover, in cells with 9 days or longer time of α-syn induction, nutrient deprivation can’t enhance autophagy any more.

### Nutrient deprivation promotes accumulation of α-syn assemblies and caspase activation in cells with induced α-syn expression

**Figure 5:**
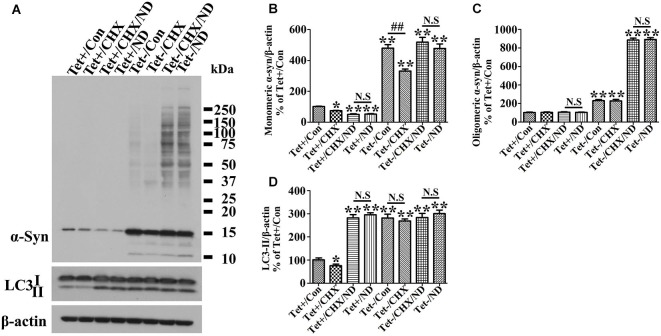
**Inhibition of protein synthesis by cycloheximide does not counteract nutrient deprivation induced degradation or aggregation of α-syn in 3D5 cells**. RA-elicited differentiated 3D5 cells with or without TetOff induction for 10 days, regarded as Tet (−) and Tet (+) (i.e., control), were respectively subjected to cycloheximide (CHX, 20 µM), nutrient deprivation or both for 24 h. Sibling cells without nutrient deprivation were used as controls. **(A)** Lysates were probed with antibodies to α-syn, LC3, β-actin by western blotting. **(B,C,D)** Bar graphs summarized quantitative analysis of the immunoreactivities of α-syn, LC3-II from three independent experiments and normalized with β-actin immunoreactivities. The average values of Tet+/Con group were set as 100%. Error bars represent standard error of the mean (* *p* < 0.05, ** *p* < 0.01, comparing to Tet+/Con; N.S., no significant difference comparing subsets linked by line).

Since α-syn is predominantly degraded through autophagic-lysosomal pathway, autophagy activation is expected to facilitate α-syn clearance. Indeed our studies of cultures expressing only endogenous α-syn (i.e., non-induced) showed nutrient deprivation lowered the levels of monomeric α-syn in non-induced cells (Figures 2A,B) without affecting cell viability (Figure 2P). Regardless of the nutrient state of culturing media, oligomeric α-syn products were not evidently detected in non-induced cells (Figures 2A,C). In comparison, α-syn products of various sizes were readily detected in cell cultures bearing more than 9 days of induced α-syn, and the amount increased further under nutrient-deprived condition (Figures 2A, 3A). Interestingly, cell cultures with 9 days or longer durations of induction in nutrient deprived media displayed significantly more α-syn oligomers (about 4 times) than counterparts in complete media (Figures 2C, 3C). Therefore, the effects of nutrient deprivation on α-syn protein levels in cells with and without overexpressed α-syn were different. We supposed that such difference might be due to the alteration of transcription, synthesis or degradation of α-syn in response to nutrient deprivation. To clarify the exact mechanism underlying the above difference, cells with and without 10 days of α-syn induction were subjected to complete or B27 deprived media with or without cycloheximide treatment for 24 h. The levels of α-syn transcript and protein were compared by quantitative PCR and western blotting, respectively. Results showed that in 3D5 cells with and without overexpressed α-syn, (1) nutrient deprivation did not cause significant changes at the level of α-syn transcripts (see Figure 2Q); (2) cycloheximide treatment alone for 24 h did cause the reduction of α-syn protein level to some extent (Figures 5A–C), indicating the successful inhibition of protein synthesis; and (3) however, such treatment did not prevent the respective reduction and aggregation of α-syn induced by nutrient deprivation (Figures 5A–C). Therefore, degradation or other pathway rather than transcription and protein synthesis should be involved in the changes of α-syn level induced by nutrient deprivation. It is worth noting that, consistent with previous study (Lawrence and Brown, 1993), in cells without α-syn induction and nutrient deprivation, cycloheximide can partially inhibit the formation of autophagosomes reflected by the reduction of LC3-II (Figures 5A,D). Under such condition, α-syn level still decreased (Tet+/CHX vs. Tet+/Con), which was most likely due to the combined effects of completely blocked α-syn synthesis and remained autophagic degradation. However, such inhibition of autophagy by cycloheximide didn’t happen to cells without α-syn induction but under nutrient deprivation, and those with overexpressed α-syn under either control or nutrient deprived condition. We believed this was because that the effect of nutrient deprivation or overexpressed α-syn on autophagy activation were strong enough to counteract inhibitory effect from cycloheximide.

Besides, we noticed that α-syn aggregation in cells with 9 days or longer durations of α-syn induction under nutrient deprivation was accompanied by the change of cell viability (Figure 2P). The effects of nutrient deprivation on cell viability appeared to be more pronounced for cultures expressing higher levels of α-syn (Figure 3G).

### Nutrient deprivation induces ER stress response which is associated with α-syn aggregation

We next want to know if α-syn degradation in cells bearing high dose of α-syn is impaired by nutrient deprivation. We thereby compared the Cathepsin D activity between 3D5 cells with and without nutrient deprivation since it is the main enzyme responsible for α-syn degradation in lysosome (Sevlever et al., 2008). Results showed that in cells with and without 10 days of α-syn induction, nutrient deprivation did not significantly impair the Cathepsin D activity (Figure 2S), which indicated that other pathway(s) should be responsible for α-syn aggregation.

Previous cell-based studies have shown that deprivation of B27 from culture medium can induce ER stress (Tajiri et al., 2006), and that such stress can enhance accumulation of α-syn oligomers (Jiang et al., 2010). To test whether accumulation of α-syn products observed in our nutrient-deprived cells was associated with ER stress, cells lysates were analyzed for the levels of several ER stress markers, including p-EIF2α vs. total EIF2α, GRP78 and CHOP, and the amount of these markers in cultures with and without the deprivation were compared (Figure 2A). Consistent with previous studies (Tajiri et al., 2006), we found a small, but significant increase of these markers (compared Tet (−)/Con to Tet (+)/Con (Figures 2J,K,L)) in cultures with a 10-day induced α-syn expression. Cells with nutrient deprivation displayed significantly higher levels of p-EIF2α and GRP78 (Figures 2J,K), respectively, than counterparts without. CHOP immunoreactivities were detected mainly in α-syn-induced cells under nutrient deprivation (Figures 2A,L). In addition to CHOP, which plays a role in apoptotic cell death, we detected cleaved-caspase 3 indicative of caspase activation mainly in cells with α-syn induction plus nutrient-deprivation (Figures 2A,M). However, we did not find significant difference in the profile of caspase 12 between them (Figure 2H), indicating that activation of caspase 12 was not responsible for the above changes.

We also examined GRP78 expression level in 3D5 cells with various durations of induced α-syn expression and maintained either in nutrient deprived or complete medium. In general, cells with longer periods of α-syn induction displayed more GRP78 (Figures 3A,E), and higher levels of GRP78 were detected in α-syn induced cells under nutrient deprivation (Figures 3A,E). Comparing to cells bearing no induced α-syn with or without nutrient deprivation (Tet(+) or Tet (+)/nutrient deprivation), 3 days of α-syn induction cannot induce significant increase of GRP78 level, such increase can only be found in cells with 6 or more days of induction, indicating that 3D5 cells can handle the presence of certain amounts of α-syn without eliciting of ER stress, and once the level of α-syn reaches a threshold, ER stress response occurs. On the other hand, nutrient deprivation induced α-syn aggregation can only be found in 6 days or longer time of α-syn induction, indicating that a critical concentration of α-syn is also needed for its aggregation induced by nutrient deprivation.

**Figure 6:**
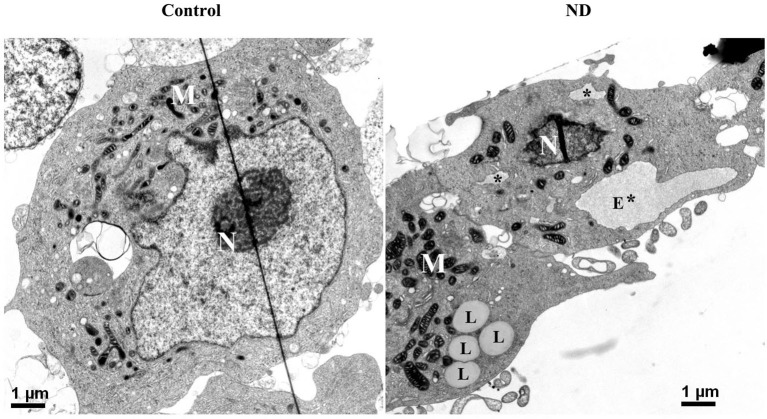
**Electron micrographs of differentiated 3D5 cells with and without nutrient deprivation**. 3D5 cells with RA-elicited differentiation and 12 days of TetOff induction were subjected to nutrient deprivation for 24 h. Sibling cell cultures without the deprivation were used as control. Cells were preserved with a fixative and prepared for ultrastructural evaluation. Nutrient-deprived cells (ND) were shown to contain structures resembling swollen/enlarged ER (labeled with E* for big lumen or * for smaller one), which were absence in cells maintained in complete media (Con). Lipid droplets clusters were detected more easily in cells with nutrient deprivation (labeled with L) than without. Nucleus was labeled with N, and mitochondria clustering area was labeled with M. Scale bar: 1 µm.

Besides probing cell lysates with ER stress markers, we used electron microscopy to examine RA-differentiated 3D5 cells with 12 days of induced α-syn expression plus nutrient deprivation or not. In comparison to cells without nutrient deprivation, those under nutrient deprivation contained abnormal subcellular organelles consistent with swollen/enlarged ER in morphology (marked with E* or * in Figure 6), as well as subcellular organelles resembling lipid droplets (marked with L, Figure 6).

Therefore, nutrient deprivation significantly induces ER stress response in 3D5 cells. To determine whether or not such ER stress response is associated with α-syn aggregation, we alleviated it by overexpressing GRP78 in 3D5 cells and found that nutrient deprivation triggered α-syn aggregation can be significantly reduced (see Figures 8A,B,C,K,L), indicating that ER stress response plays a key role (see more details in next section).

**Figure 7:**
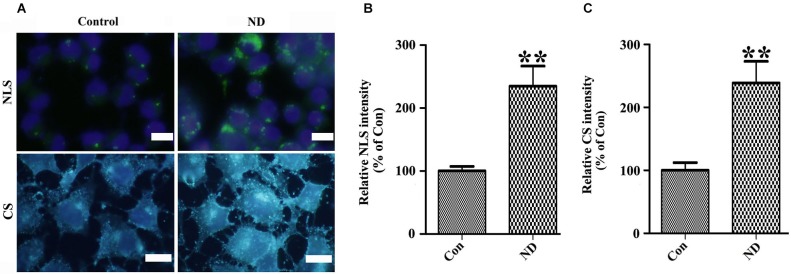
**Nutrient deprivation induces accumulation of intracellular neutral lipids and cholesterol in 3D5 cells**. 3D5 cells with RA-elicited differentiation and 10 days of TetOff induction were subjected to nutrient deprivation for 24 h. Sibling cell cultures without the deprivation were used as control. Cells were fixed in 4% paraformaldehyde and stained with **(A)** LipidTOX™ Green and Filipin for evaluation of neutral lipids and cholesterol, respectively. NLS: Neutral lipids staining; CS: Cholesterol Staining. Scale bar: 5 µm. **(B,C)** Results from quantitative image analysis using ImageJ demonstrated that nutrient deprivation induced significant increases of both intracellular neutral lipids **(B)** and cholesterol **(C)** contents. Error bars represent standard error of the mean (** *p* < 0.01, *n* = 3).

**Figure 8:**
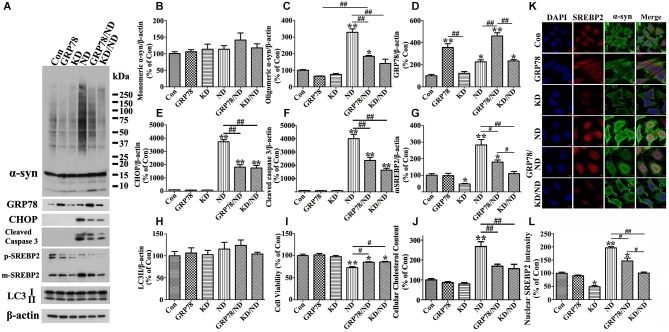
**Overexpression of GRP78 or knockdown of SREBP2 reduces α-syn aggregation in 3D5 cells elicited by nutrient deprivation**. 3D5 cells with 7 days RA-elicited differentiation and Tetoff induced α-syn overexpression were infected with lentivirus carrying empty vector (Con), GRP78, or shRNA of human SREBP2 (KD) as described in the Materials and Methods section. 4 days past infection, a subset of each cell group was maintained in complete media and referred to as Con, GRP78 and KD, respectively, while another subset was subjected to nutrient deprivation for 24 h and referred to as ND, GRP78/ND and KD/ND. Duplicated cultures were included for cell viability assessment and cholesterol content measurement. **(A)** Cell lysates were probed with antibodies to α-syn, GRP78, CHOP, cleaved Caspase 3, SREBP2, LC3 and β-actin. Molecular weight standards were included as references. **(B–H)** Bar graphs summarized quantitative immunoblot analyses of various proteins from three independent experiments and normalized with β-actin. The average values of Con group were set as 100%. **(I)** Calcein assay showed that GRP78 overexpression or SREBP2 knockdown can significantly rescue cell loss caused by nutrient deprivation. **(J)** Amplex Red-based cholesterol assay showing GRP78 overexpression or SREBP2 knockdown can prevent nutrient deprivation-induced cholesterol increase in 3D5 cells. Error bars represent standard error of the mean (* *p* < 0.05, ** *p* < 0.01, comparing to Con; # *p* < 0.05, ## *p* < 0.01, comparing subsets linked by line). **(K)** Immunocytochemistry demonstrated nutrient deprivation induced changes of the levels of α-syn and SREBP2 in cells of aforementioned six groups. Scale bar: 5 µm. **(L)** Bar graphs summarized quantitative analysis of nuclear SREBP2 fluorescence intensity from at least 50 immunocytochemically stained 3D5 cells per group (* *p* < 0.05, ** *p* < 0.01, comparing to Con; # *p* < 0.05, ## *p* < 0.01, comparing subsets linked by line).

### Nutrient deprivation triggered ER stress response induces SREBP2 activation and subsequent cholesterolgenesis, which are associated with α-syn aggregation

We next wondered how ER stress response plays the role in facilitating α-syn aggregation. Because (1) during cell experiments we noticed that under nutrient deprivation, cells displayed more refractive elements of size resembling lipid droplets; (2) lipid droplets-like structures can be easily observed in nutrient deprived cells under electron microscopy; (3) nutrient deprivation can increase lipid droplets in neurons and other types of cells and modulate the synthesis of lipid and cholesterol by triggering ER stress response (Werstuck et al., 2001; Lee et al., 2004; Du et al., 2009; Basseri and Austin, 2012; Cabodevilla et al., 2013; Fang et al., 2013); and (4) both lipid and cholesterol can interact with α-syn and facilitate its aggregation (Sharon et al., 2001; Bar-On et al., 2008; Fantini et al., 2011), we thereby hypothesized that nutrient deprivation triggered ER stress response might promote α-syn aggregation by increasing lipid and cholesterol content.

### Nutrient deprivation induces intracellular accumulation of neutral lipid and cholesterol as well as activation of SREBP1c and SREBP2

To confirm the above hypothesis, we first determined whether or not lipid and cholesterol content were really increased in cells upon nutrient deprivation. We stained our cultures with LipidTox™ Green and Filipin to respectively label neutral lipids and cholesterol. Results showed that 3D5 cells with nutrient deprivation displayed more intense signals of neutral lipids and cholesterol than those without (Figure 7A). Quantitative analysis of images derived from similar number of cells showed nutrient-deprived cells containing about 2~3 times more neutral lipid and cholesterol than that those maintained in complete media (Figures 7B,C).

We also probed cell lysates with antibodies specific to SREBP1 and SREBP2, since they are transcription factors function to regulate lipogenesis and cholesterol metabolism (Wang et al., 1994; Brown and Goldstein, 1997; Horton et al., 2002), respectively. Cells under nutrient deprivation were demonstrated by western blotting to contain significantly more matured forms of SREBP1 and SREBP2 than those maintained in complete medium (Figures 2A,N,O), indicative of their activation. We also did immunocytochemistry for SREBP2 in cells with and without nutrient deprivation and demonstrated that nutrient deprivation evidently induced nuclear translocation of SREBP2 (see Figures 8K,L), further confirmed the activation of SREBP2.

### Knockdown of SREBP2 but not SREBP1 prevents nutrient deprivation-induced increase of cholesterol, α-syn aggregation and caspase activation

It has been reported that α-syn interacts with lipid and cholesterol and such interactions can affect α-syn self-interactions (Sharon et al., 2001; Bar-On et al., 2008; Fantini et al., 2011). To determine whether enhanced accumulation of α-syn oligomers displayed in our nutrient-deprived cultures was caused by increase of lipid and/or cholesterol, cells with either SREBP1 or SREBP2 knockdown were generated and counterparts without the knockdown served as controls. Western blotting and immunocytochemistry were respectively used to confirm the successful silence of SREBPs (see Figures 8A,G,K,L). Moreover, we found that knockdown of SREBP2 (Figure 8G) can significantly prevent cells from nutrient deprivation induced α-syn aggregation (Figure 8C), increase of CHOP (Figure 8E) and caspase 3 activation (Figure 8F), and loss of viability without affecting LC3-II levels (Figure 8H), but had no much effect on the above parameters in cells without nutrient deprivation. However, SREBP1 knockdown was cytotoxic because it almost kills all cells after 24 h of nutrient deprivation (data no shown). Therefore, we only focused on the effect of SREBP2 and its related cholesterol content in present study. Consistent with the known function of SREBP2, the levels of cholesterol were lower in cells with SREBP2 knockdown than those without (Figure 8J).

**Figure 9:**
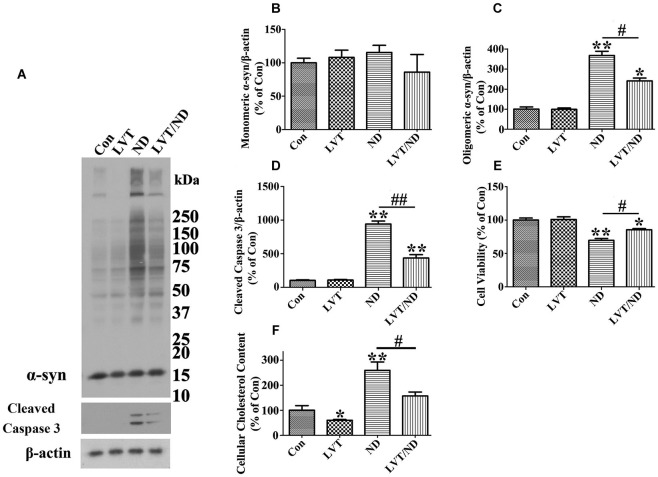
**Lovastatin treatment partially prevents cultured cells from α-syn oligomer accumulation and cell death elicited by nutrient deprivation**. RA-elicited differentiated 3D5 cells with TetOff induction for 12 days were subjected to treatment with DMSO [regarded as control (Con)], 5 µM lovastatin (LVT), DMSO plus nutrient deprivation, or 5 µM LVT plus nutrient deprivation (LVT/ND) for 24 h. **(A)** Cell lysates were probed with antibodies to α-syn, cleaved caspase 3 and β-actin. Molecular weight standards were included as references. **(B–D)** Bar graphs summarized quantitative immunoblot analysis of various proteins from three independent experiments with normalization against β-actin immunoreactivities. The average values of Con group were set as 100%. **(E)** Calcein assay of duplicated cultures showed that LVT treatment can partially prevent the cell loss caused by nutrient deprivation. **(F)** Amplex Red-based cholesterol assay showed that cholesterol levels in cells with or without nutrient deprivation were significantly reduced upon LVT pre-treatment. Error bars represent standard error of the mean (* *p* < 0.05, ** *p* < 0.01, comparing to Con; # *p* < 0.05, ## *p* < 0.01, comparing subsets linked by line). The results are representative of three independent experiments.

To clarify whether or not cholesterol is a key factor responsible for α-syn oligomers accumulation under nutrient deprivation, cell cultures overexpressing α-syn were pretreated with lovastatin, an agent known to decrease cholesterol levels (Alberts, 1988), followed by exposure to nutrient deprivation. Sibling cultured cells without the pretreatment were included as control. Our measurement of cholesterol confirmed the effect of lovastatin on cholesterol reduction (Figure 9F). Western blotting of cell lysates showed that lovastatin treatment significantly reduced oligomeric α-syn accumulation, caspase activation and cell loss elicited by nutrient deprivation (Figures 9A,C,D,E). These results supported an important role of cholesterol in facilitating α-syn aggregation under nutrient deprivation.

### Alleviation of ER stress response by GRP78 overexpression prevents nutrient deprivation induced SREBP2 activation, cholesterol increase, α-syn aggregation and cell apoptosis

Next, we want to know whether or not ER stress response is responsible for SREBP2 activation, increase of cholesterol content and accumulation of α-syn oligomers observed in nutrient-deprived cells. For this purpose, we infected 3D5 cells with lentivirus carrying either GRP78 or empty vector, and subsequently subjected these cells to nutrient deprivation. Cultures infected with lentivirus carrying GRP78 displayed significantly more GRP78 and less α-syn oligomers than those infected with empty vector (Figures 8A,C,D compared GRP78/ND to ND), even though the level of oligomers did not revert to that displayed in cultures without nutrient deprivation (Compared GRP78/ND to Con in Figures 8A,C). Under nutrient-deprived condition cells with GRP78 overexpression also displayed significantly lower levels of CHOP, activated SREBP2 and activated caspase 3 than those without (Figures 8A–G). Furthermore, our assays of cell viability and cholesterol levels showed a protective effect of GRP78 overexpression on cell loss and elevation of cholesterol levels caused by nutrient deprivation (Figures 8I–J). Thus, the results together with those derived from SREBP2 knockdown and lovastatin studies supported a strong link among α-syn oligomers accumulation, SREBP2 activation, increase of cholesterol levels and ER stress response, and suggested that nutrient deprivation triggered ER stress response induces SREBP2 activation and subsequent cholesterolgenesis, which are responsible for observed α-syn aggregation.

### Alpha-syn overexpression affects primary neuronal cultures and 3D5 cells similarly in their response to nutrient deprivation

**Figure 10:**
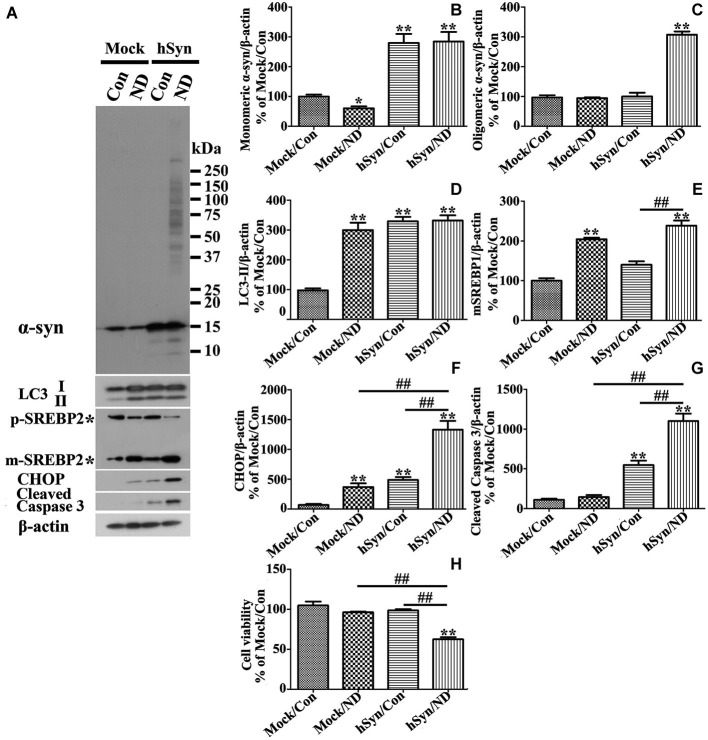
**Primary neuronal cultures with α-syn overexpression differ from those without in response to nutrient deprivation**. Primary neuronal cultures were infected with empty vector (Mock) and human α-syn (hSyn) and maintained in media with nutrient deprivation (ND) or without (Con) for 24 h. **(A)** Cell lysates were probed with antibodies to α-syn, LC3, SREBP2, CHOP, cleaved Caspase 3, and β-actin by western blotting. Molecular weight standards were included as references. Duplicate cultures were included for cell viability assessment. **(B–G)** Bar graphs summarized quantitative analysis of immunoblots of proteins of interest from three independent experiments and normalized with β-actin immunoreactivities. The average values of Mock/Con were set as 100%. **(H)** Calcein assay showed that nutrient deprivation caused a significant lose of viability in cells overexpressing human α-syn, but not in those without. Bar graph summarized the data from three independent experiments. Error bars represent standard error of the mean (* *p* < 0.05, ** *p* < 0.01, comparing to Mock/Con; # *p* < 0.05, ^##^ *p* < 0.01, comparing subsets linked by line).

To determine whether the results obtained from studies of 3D5 cultures are applicable to other types of culture we used primary neuronal cultures infected with lentivirus carrying human α-syn or empty vector as control. These cultures were subjected to 24 h of nutrient deprivation or not. Similar to those observed in non-induced 3D5 cells, nutrient deprivation of primary cultures infected with empty vector caused a significant decrease of monomeric α-syn as well as increase of LC3-II and SREBP2 without a significant effect on caspase 3 activation and cell viability (Figures 10A,B,D,E,G,H). In contrast to that demonstrated by 3D5 cells, without α-syn overexpression nutrient deprivation of primary cultures caused a small, but significant increase of CHOP (Figure 10F), suggesting they are more sensitive to nutrient deprivation. Similar to 3D5 cells with Tetoff induction, α-syn overexpression rendered primary neuronal cultures to increase the level of LC3-II without significant activating SBERP2. It is worth noting that the overexpressed α-syn is about 3 fold of its endogenous level (Figures 10A,B). Moreover, in these cultures, nutrient deprivation also induced higher levels of active caspase 3 and CHOP (Figures 10G,F), lower viability (Figures 10A,F,H) without affecting LC3-II levels (Figures 10A,D).

**Figure 11:**
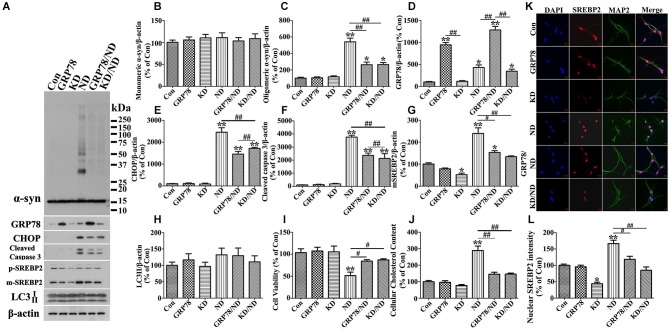
**GRP78 overexpression or SREBP2 knockdown reduces α-syn aggregation in primary neuronal cultures elicited by nutrient deprivation**. Primary cultures with human α-syn overexpression were infected with lentivirus carrying GRP78 or shRNA of mouse SREBP2. 7 days after the infection, cells were maintained in media with or without nutrient deprivation for 24 h. Duplicated cultures in 48 well plates were included for cell viability assessment and cholesterol assay. **(A)** Cell lysates were probed with antibodies to α-syn, GRP78, CHOP, cleaved Caspase 3, SREBP2 and β-actin. Molecular weight standards were included as references. **(B–H)** Bar graphs summarized quantitative immunoblot analysis of various proteins from three independent experiments and normalized with β-actin. The average values of Con groups were set as 100% for all other proteins. **(I)** Calcein assay showing overexpression of GRP78 or knockdown of SREBP2 significantly rescued cell loss elicited by nutrient deprivation. **(J)** Amplex Red-based cholesterol assay showing GRP78 overexpression or SREBP2 knockdown can prevent nutrient deprivation-induced cholesterol increase in primary cultures. Error bars represent standard error of the mean (* *p* < 0.05, ** *p* < 0.01, comparing to Con; # *p* < 0.05, ## *p* < 0.01, comparing subsets linked by line). **(K)** Immunocytochemistry demonstrated the alteration of SREBP2 level in primary neurons (showing neuronal staining with MAP2) of aforementioned six groups. Scale bar: 10 µm. **(L)** Bar graphs summarized quantitative analysis of nuclear SREBP2 fluorescence intensity from at least 50 immunocytochemically stained primary neurons per group (* *p* < 0.05, ** *p* < 0.01, comparing to Con; # *p* < 0.05, ^##^ *p* < 0.01, comparing subsets linked by line).

We also tested whether SREBP2 knockdown or GRP78 overexpression can affect primary neuronal cultures overexpressing human α-syn in a manner comparable to that demonstrated by 3D5 cells. Western blotting and immunocytochemistry were respectively performed to confirm successful silence of SREBP2 in primary neurons (see Figures 11A,G,K,L). These genetic manipulations were shown to reduce the impact of nutrient deprivation on the levels of α-syn oligomers, SREBP2, active caspase 3, CHOP, LC3-II and cell viability (Figure 11) in primary cultures as same as in 3D5 cells.

## Discussion

In present study, we demonstrated that nutrient deprivation resulted in no reduction but a mark increase of α-syn oligomers accompanied by decrease of cell viability in neuron cells with 10 days of α-syn induction. These are in contrast to the results obtained from those bearing only endogenous α-syn, which under nutrient deprivation displayed significantly less α-syn monomers since no α-syn oligomers can be detected (Figures 2, 3). Besides, nutrient deprivation caused a significantly increased levels of autophagy markers in cells without α-syn induction, but did not affect them in cells with overexpressed α-syn (see Figures 2, 3). Further study demonstrated that a 3-day induced α-syn overexpression alone caused increase of LC3-II to a level about 1.5 times of that in non-induced control (Figure 3). By 9 days of induction, the level of LC3-II is about the same as that expressed in non-induced cultures with nutrient deprivation. At this time point, the level of monomeric α-syn is about 5~6 times of that displayed by non-induced cells (Figure 3). Our analysis of autophagic flux showed induction for 9 days or more led to increase of autophagic flux to a level near that in non-induced cultures with nutrient deprivation (Figure 4). Thus, high levels of α-syn overexpression did not appear to suppress autophagy. Instead, it activates autophagy to an extent comparable to that achieved by nutrient deprivation. Nutrient-deprived 3D5 cells bearing high level of α-syn (i.e., 9 days of induction or more) are comparable to non-deprived counterparts in the level of LC3-II and autophagic flux, suggesting these cells have reached their maximum capacity in autophagy so that nutrient deprivation could not enhance autophagy to accelerate α-syn degradation as effectively as it could in cells without α-syn induction. It is worth noting that α-syn overexpression has been shown to activate or impair autophagy in different studies (Winslow et al., 2010; Choubey et al., 2011; Wong et al., 2011). Based on the results obtained from our cell models, we supported the role of α-syn in autophagy activation.

The induction time-dependent increase of oligomeric α-syn could be due to increase of α-syn monomer for self-assembly. This alone, however, is not sufficient to explain how or why nutrient-deprived cells have marked increase of α-syn oligomers because (1) nutrient deprivation did not cause significant changes at the level of α-syn transcripts (Figure 2Q); and (2) inhibition of protein synthesis by cycloheximide did not prevent α-syn aggregation triggered by nutrient deprivation (Figure 5). Another possibility might be that α-syn degradation is impaired due to lysosome dysfunction caused by nutrient deprivation, which were also ruled out by the fact that Cathepsin D activities have no significant difference (Figure 2S) and the level of autophagy flux remains no change in cells from different groups. Therefore, we hypothesized that factor(s) other than impaired degradation elicited under nutrient deprivation is involved and it can act as a promoter for α-syn assembly.

We noted that under nutrient-deprivation 3D5 cells differed from non-deprived counterparts by containing abnormal structures resembling swollen ER, and increased amount of retractile elements resembling lipid droplets (Figure 6). We also demonstrated that the nutrient-deprived cultures contained larger amounts of neutral lipids, cholesterol and ER stress markers (Figures 2, 7). These changes were associated with increase of activation of SREBP1 and SREBP2, which are important for lipogenesis and cholesterolgenesis (Horton et al., 2002). Therefore, it seems reasonable to assume that enhanced accumulation of α-syn oligomers in nutrient-deprived cells is due to the increase of lipids/cholesterol caused by ER stress response, since α-syn self-interactions can be modulated by presence of lipid/cholesterol (Sharon et al., 2001; Bar-On et al., 2008; Fantini et al., 2011), and synthesis of these molecules can be affected upon ER stress response (Colgan et al., 2007).

To confirm the above hypothesis, we first proved that nutrient deprivation induced α-syn aggregation is regulated by SREBP2 activation and subsequent increased cholesterolgenesis. It is worth noting that knockdown of SREBP1 was not protective but harmful, which might be due to the blockade of lipogenesis under nutrient deprivation because lipid metabolism is critical pathway to the survival of starved cells (Cabodevilla et al., 2013). On the other hand, the cholesterol lowering agent—lovastatin effectively protected cells from increase of α-syn oligomers, CHOP, caspase activation and cell loss caused by nutrient deprivation (Figure 9). More importantly, SREBP2 knockdown did show significant protection as shown in lovastatin, indicating a close link between cholesterol level and α-syn aggregation. In this regard, a recent report showed that SREBP2 transgenic mice exhibited early mitochondrial cholesterol loading and mitochondrial glutathione depletion, indicating the toxic effect of SREBP2 overexpression (Barbero-Camps et al., 2013). In support of this study, our results further suggested that down-regulation of SREBP2 activity might be useful to reduce cholesterol level for preventing α-syn toxicity in PD brain since high level of cholesterol is tightly related to α-syn aggregation in PD (Bosco et al., 2006).

Next, we further confirmed that nutrient deprivation induced SREBP2 activation, cholesterol increase, α-syn aggregation and cell apoptosis can be rescued by alleviation of ER stress via overexpression of GRP78, which is a HSP70 molecular chaperon located in the lumen of ER that binds newly synthesized proteins and maintained them in a state competent for subsequent folding (Haas, 1994). Our results are consistent with those obtained in previous studies, in which HSP70 overexpression has a protective effect on α-syn cytotoxicity and oligomer accumulation (Klucken et al., 2004). More importantly, a consequence of GRP78 overexpression in 3D5 is decrease of active form of SREPB2 in the nutrient-deprived (Figures 8G,K,L). On the other hand, other researchers have reported that silencing SREBPs can increase ER stress (Choi et al., 2011), which did not happen in our experiment. We think this is because (1) our study was done under a completely different scenario—nutrient deprivation; and (2) our cell types are neuronal cells which are different from theirs.

The impacts of nutrient-deprivation observed in 3D5 cells are not unique to one culture type. Our studies also showed that the response of primary neuronal cultures to nutrient deprivation in a manner comparable to those demonstrated in 3D5 cells (Figures 10, 11). It remains to be tested whether obtained from our studies are applicable to neuronal cells *in vivo*, particularly those in with SCNA gene multiplication or older subjects, since α-syn level has been reported to increase with aging (Jellinger, 2004; Li et al., 2004; Chu and Kordower, 2007). Regardless, it is worth noting that the state of preexisting activated autophagy and reduced responsiveness caused by high level of α-syn in neurons of PD brain should be considered when the potential therapeutic agents aiming at activating autophagy for PD therapy are developed.

In summary, our study showed (i) α-syn overexpression activates autophagy in neurons cells; (ii) nutrient deprivation did not enhance autophagy in cells bearing high level of α-syn; (iii) nutrient deprivation promoted α-syn aggregation and cell apoptosis by triggering ER stress response in which SREBP2 activation and its function on cholesterolgenesis were involved; (iv) these changes were reduced substantially by reducing cholesterolgenesis; and (v) in this regard, down-regulation of SREBP2 activity might be a means to prevent α-syn aggregates accumulation in PD.

## Conflict of interest statement

The authors declare that the research was conducted in the absence of any commercial or financial relationships that could be construed as a potential conflict of interest.
